# *TREM2* is associated with increased risk for Alzheimer’s disease in African Americans

**DOI:** 10.1186/s13024-015-0016-9

**Published:** 2015-04-10

**Authors:** Sheng Chih Jin, Minerva M Carrasquillo, Bruno A Benitez, Tara Skorupa, David Carrell, Dwani Patel, Sarah Lincoln, Siddharth Krishnan, Michaela Kachadoorian, Christiane Reitz, Richard Mayeux, Thomas S Wingo, James J Lah, Allan I Levey, Jill Murrell, Hugh Hendrie, Tatiana Foroud, Neill R Graff-Radford, Alison M Goate, Carlos Cruchaga, Nilüfer Ertekin-Taner

**Affiliations:** Department of Psychiatry, Washington University School of Medicine, 660 South Euclid Avenue B8134, St. Louis, MO 63110 USA; Department of Neuroscience, Mayo Clinic, 4500 San Pablo Road, Jacksonville, FL 32224 USA; Departments of Neurology, Psychiatry and Epidemiology, Columbia University, New York, NY 10032 USA; Taub Institute for Research on Alzheimer’s Disease and the Aging Brain, College of Physicians and Surgeons, Columbia University, New York, NY 10032 USA; Department of Neurology, Emory University School of Medicine, Atlanta, GA 30033 USA; Division of Neurology, Atlanta Veterans Administration Medical Center, Atlanta, GA 30033 USA; Department of Pathology and Laboratory Medicine, Indiana University School of Medicine, Indianapolis, IN 46202 USA; Indiana University Center for Aging Research, Indianapolis, IN 46202 USA; Regenstrief Institute, Inc, 410 West 10th Street, Suite 2000, Indianapolis, IN 46202 USA; Department of Medical and Molecular Genetics, Indiana University School of Medicine, Indianapolis, IN 46202 USA; Department of Neurology, Mayo Clinic, 4500 San Pablo Road, Jacksonville, FL 32224 USA; Hope Center Program on Protein Aggregation and Neurodegeneration, Washington University School of Medicine, 660 S. Euclid Avenue B8111, St. Louis, MO 63110 USA; Department of Neurology, Washington University School of Medicine, 660 S. Euclid Avenue B8111, St. Louis, MO 63110 USA; Joanne Knight Alzheimer’s Disease Research Center, Washington University School of Medicine, 4488 Forest Park Avenue, St. Louis, MO 63108 USA; Department of Genetics, Washington University School of Medicine, 660 S. Euclid Avenue, St. Louis, MO 63110 USA

**Keywords:** LOAD, African-American, TREM2, Coding variants, Case–control

## Abstract

**Background:**

*TREM2* encodes for triggering receptor expressed on myeloid cells 2 and has rare, coding variants that associate with risk for late-onset Alzheimer’s disease (LOAD) in Caucasians of European and North-American origin. This study evaluated the role of *TREM2* in LOAD risk in African-American (AA) subjects. We performed exonic sequencing and validation in two independent cohorts of >800 subjects. We selected six coding variants (p.R47H, p.R62H, p.D87N, p.E151K, p.W191X, and p.L211P) for case–control analyses in a total of 906 LOAD cases vs. 2,487 controls.

**Results:**

We identified significant LOAD risk association with p.L211P (p = 0.01, OR = 1.27, 95%CI = 1.05-1.54) and suggestive association with p.W191X (p = 0.08, OR = 1.35, 95%CI = 0.97-1.87). Conditional analysis suggests that p.L211P, which is in linkage disequilibrium with p.W191X, may be the stronger variant of the two, but does not rule out independent contribution of the latter. *TREM2* p.L211P resides within the cytoplasmic domain and p.W191X is a stop-gain mutation within the shorter TREM-2V transcript. The coding variants within the extracellular domain of TREM2 previously shown to confer LOAD risk in Caucasians were extremely rare in our AA cohort and did not associate with LOAD risk.

**Conclusions:**

Our findings suggest that *TREM2* coding variants also confer LOAD risk in AA, but implicate variants within different regions of the gene than those identified for Caucasian subjects. These results underscore the importance of investigating different ethnic populations for disease risk variant discovery, which may uncover allelic heterogeneity with potentially diverse mechanisms of action.

**Electronic supplementary material:**

The online version of this article (doi:10.1186/s13024-015-0016-9) contains supplementary material, which is available to authorized users.

## Background

TREM2 is a transmembrane receptor expressed on microglia, macrophages, monocyte-derived dendritic cells, and osteoclasts [1,2]. In the human brain, TREM2 is primarily expressed on microglia and controls two signaling pathways: regulation of phagocytosis and suppression of inflammatory reactivity. Previous studies have shown that homozygous loss-of-function mutations in *TREM2* are associated with the polycystic lipomembranous osteodysplasia with sclerosing leukoencephalopathy (PLOSL; also called Nasu-Hakola disease) characterized by progressive early-onset dementia and bone lesions leading to fractures [3-5]. Association between *TREM2* and Alzheimer’s disease (AD) was initially reported by two independent groups, who identified a rare heterozygous missense mutation in *TREM2*, p.R47H (rs75932628-T), that increased AD risk by about 2–4.5 fold [6,7]. One of these studies also reported additional, rare coding variants in exon 2 of *TREM2* that were collectively enriched in AD cases compared to controls [6].

Since then, association between p.R47H and AD risk was replicated in Spanish and French-Caucasian populations [8-10] and other North American-Caucasian series, [11,12] but not in Asian populations, where this variant is either very rare or absent, highlighting the need to study diverse populations [13,14]. Several studies suggest that *TREM2*-p.R47H could also be a risk factor for Parkinson’s disease, frontotemporal dementia (FTD), and amyotrophic lateral sclerosis [15-18]. These observations clearly highlight two common themes in neurodegenerative diseases: (1) homozygous and heterozygous mutations in *TREM2* have pleiotropic effects on clinically distinct disorders and (2) neuroinflammation plays a key role in neurodegenerative disease pathogenesis.

To identify additional AD risk variants in *TREM2*, several deep re-sequencing studies have been performed to comprehensively screen the coding regions of this gene. Cuyvers et al. sequenced *TREM2* in a Belgian population and found coding variants [19], some of which were not previously reported [6]. Although none of the observed variants were significantly associated with AD risk, Cuyvers et al. found an enrichment of *TREM2* variants in both AD and FTD patients compared to controls [19]. Recently, our group performed deep re-sequencing of *TREM2* coding regions in approximately 4,000 individuals of European descent [20]. We identified sixteen non-synonymous variants, six of which were not identified in previous AD studies [20]. Besides p.R47H, we found that p.R62H was significantly associated with AD risk.

Despite increasing numbers of reports on *TREM2* associations in subjects of European descent, to our knowledge, there are no comprehensive studies of *TREM2* coding variants in African-Americans (AAs). Furthermore, to date, no deep re-sequencing studies have been conducted to comprehensively catalogue *TREM2* rare variants in AAs. Given this knowledge gap and notable differences of AD risk variants between AAs vs. Caucasians [21], assessment of *TREM2* in this ethnic group can bring novel insight into the role of this gene in AD. In this study, we hypothesized that coding *TREM2* variants also affect AD risk in AAs and sought to catalogue such variants in AAs. To test this hypothesis, we used targeted next-generation sequencing of pooled-DNA and Sanger sequencing of individual DNA samples to search for *TREM2* variants in two independent AA cohorts. Follow-up genotyping of six potentially functional variants was conducted in a large late-onset AD (LOAD) case–control series comprised of >3,300 AA subjects from five institutions. Finally, we conducted a joint analysis to evaluate the effect of *TREM2* variants on AD risk.

## Results

**Figure 1:**
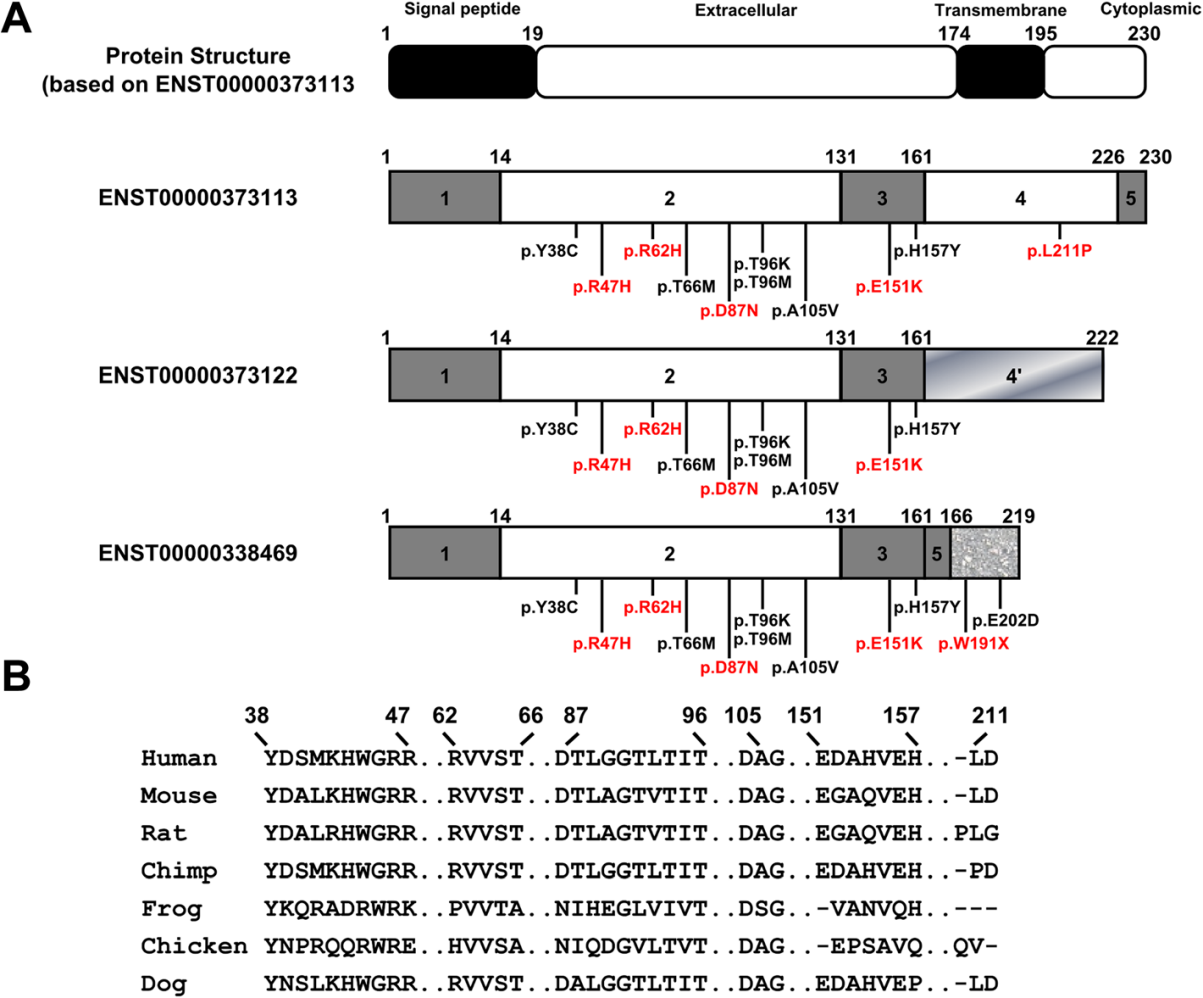
**Protein structures and conservation of TREM2 with marked variants. (A)** The top panel shows the protein structure of TREM2 (based on ENST00000373113), a type-I transmembrane receptor that is encoded by a gene containing 5 exons. The isoform ENST00000373122 encodes a different protein coding sequence after exon 3 (gradient fill rectangle) compared to ENST00000373113. The soluble form of TREM2 (ENST00000338469) lacks exon 4, which encodes the transmembrane domain, and contains a coding region after exon 5 (texture fill rectangle). Figures shown below include the structure of three different *TREM2* transcripts, the location of confirmed variants in the most common *TREM2* transcript (ENST00000373113), and the location of confirmed variants only in the *sTREM2* transcript (ENST00000338469). Most of the variants in the transmembrane form of TREM2 are located in the extracellular domain with three exceptions, located in the cytoplasmic tail. We identified two variants that are located near the C-terminus of the soluble form of TREM2. **(B)** The protein conservation analysis of confirmed *TREM2* variants. Variants are shown with an arrow identifying the corresponding amino acid position. Protein sequences were downloaded from UniProt. The entries used for each species are as follows: Q9NZC2 (human), Q99NH8 (Mouse), D3ZZ89 (Rat), H2QSZ0 (Chimp), F7CW35 (Frog), Q2YHU4 (Chicken), and E2RP46 (Dog).

Pooled sequencing in 202 AD cases and 136 controls from WUSM identified eleven non-synonymous *TREM2* variants in this AA cohort (Additional file 1: Table S7 and Figure 1A), one of which (p.A105V) has not been identified in previous AD studies [6,19,20]. Sanger sequencing of an independent AA cohort from Mayo, followed by direct genotyping in 179 AD cases and 334 controls identified seven non-synonymous *TREM2* variants (Additional file 1: Table S8 and Figure 1A). In these sequenced samples, we identified one AD subject heterozygous for p.T66M. This variant was previously identified in its homozygous state in the proband of a Turkish pedigree with an FTD-like syndrome [16] and also observed in its heterozygous state in a different Caucasian AD subject [6].

Based on the sequencing data from the WUSM and Mayo datasets, we selected six variants for follow-up genotyping in additional cohorts (Table 1): All variants were non-synonymous coding variants that were observed in both cohorts, except p.D87N, which was only observed in WUSM, but included as it was previously shown to be associated with AD risk [6]. Of the selected variants, p.R47H and p.R62H have been identified as risk factors for AD in previous studies [6,7,20]. Although p.T96K was observed in both sequenced cohorts, this was not chosen for follow-up genotyping due to its almost perfect linkage disequilibrium (LD) with p.L211P (D’ = 0.99, R^2^ = 0.96), which did not have the polyallelic variability problem of the p.T96 locus (p.T96K/M/R).

**Table 1:** **Results of the joint-analysis of 6 follow-up SNPs in** ***TREM2***

**Variant**	**SNP**	**Position**	**AD cases**			**Controls**			**p**	**OR (95% CI)**	**Missing (%)**	**SIFT**	**PolyPhen-2**
			**No. of cases**	**No. of carriers**	**MAF (%)**	**No. of controls**	**No. of carriers**	**MAF (%)**					
p.R47H	rs75932628	6:41129252	899	2	0.11	2,471	3	0.06	0.61	1.83 (0.31-10.98)	0.7	Tolerated	Damaging
p.R62H	rs143332484	6:41129207	899	3	0.17	2,476	11	0.22	1	0.75 (0.20-2.69)	0.5	Tolerated	Benign
p.D87N	rs142232675	6:41129133	888	1	0.06	2,473	2	0.04	1	1.39 (0.13-15.37)	0.9	Tolerated	Damaging
p.E151K	rs79011726	6:41127561	867	6	0.35	2,467	7	0.16	0.22	2.14 (0.74-6.17)	1.7	Tolerated	Damaging
p.W191X	rs2234258	6:41126429	884	68	3.96	2,475	145	2.97	0.08‡	1.35 (0.97-1.87)	1	NA	NA
p.L211P	rs2234256	6:41126655	888	210	12.67	2,472	529	11.1	0.01‡	1.27 (1.05-1.54)	1	Tolerated	Benign

These analyses only used samples from Washington University, Mayo Clinic, Indiana University, WHICAP, and Emory University. Positions are relative to the human genome build GRCh37. The Fisher’s exact test was used to calculate the p values and effect sizes for p.R47H, p.R62H, p.D87N, and p.E151K using PLINK. ‡Multivariate logistic regression was performed to evaluate the association of p.W191X and p.L211P with AD risk adjusting for age, gender, *APOE* genotype, and cohorts.

After completion of direct-genotyping of these variants in an additional 525 LOAD cases and 2,017 controls from Indiana, WHICAP, and Emory, we performed association analyses for *TREM2* variants in the combined cohort of 906 AA LOAD cases and 2,487 elderly AA controls. The primary results of the combined analysis of all cohorts are shown in Table 1, and analyses of individual cohorts are depicted in Additional file 1: Tables S7, S8 and Additional file 2: Tables S9, S10 and S11. Surprisingly, we did not find any significant association for the previously reported risk variants p.R47H (p = 0.61, odds ratio [OR] = 1.83 [0.31-10.98]) and p.R62H (p = 1, OR = 0.75 [0.20-2.69]) (Table 1), possibly due to their rarity in AAs. Likewise, the rare variant p.D87N was not significant in this study (p = 1, OR = 1.39 [0.13-15.37]). In contrast, p.L211P in exon 4 of *TREM2* is significantly associated with increased risk of AD (p = 0.01, OR = 1.27 [1.05-1.54]; Table 1). Additionally, p.W191X, a stop-gain variant that is only present in the shortest transcript of *TREM2*, shows a trend toward association with AD risk (p = 0.08, OR = 1.35 [0.97-1.87]; Table 1). We did not observe any significant heterogeneity in the disease association of p.W191X and p.L211P between cohorts (P_Breslow-Day_ = 0.95 and 0.25 for p.W191X and p.L211P respectively; Additional file 3: Figures S1 and Additional file 4: Figure S2). *TREM2* p.L211P and p.W191X are in tight LD but only weakly correlated (D’ = 0.98, R^2^ = 0.25). When we adjusted for p.L211P, the association of p.W191X became insignificant (p = 0.63, OR = 1.10 [0.75-1.61]; Additional file 5: Table S12). Nonetheless, after adjusting for p.W191X, p.L211P remained significantly associated with AD risk (p = 0.05, OR = 1.25 [1.00-1.57]; Additional file 5: Table S12). This suggests that of these two polymorphisms p.L211P is more likely to be the underlying risk variant or the stronger proxy for another functional variant. Finally, *TREM2* p.E151K did not achieve significance in the analysis of the combined series (p = 0.22, OR = 2.14 [0.74-6.17], Table 1).

## Discussion

This is the first investigation of *TREM2* for coding variant discovery, validation and AD risk association in a sizable cohort of AAs (n = 3,393). In this study, we identified 12 *TREM2* coding region variants, six of which were genotyped in a combined cohort of 906 AA LOAD cases and 2,487 elderly AA controls. Amongst these six coding variants, two of them, p.L211P and p.W191X, showed nominally significant or suggestive association with risk of LOAD in the combined AA cohort. *TREM2* p.L211P was previously observed in two of the three LOAD risk variant discovery studies [6,19,20] performed in Caucasian subjects of European or North American origin. Based on these studies as well as the NHLBI GO Exome Sequencing Project (ESP) Exome Variant Server (EVS) [19], *TREM2* p.L211P is a rare variant in the Caucasian population with an allele frequency of ~0.14% in comparison to African-Americans that have a MAF ~ 13%. Likewise, p.W191X, which was observed only one time in the published *TREM2* sequencing studies of Caucasians [20] and has a MAF of 0.06% in the European-American (EA) cohort from EVS, has an allele frequency of ~3% in the AA controls in this study and ~4% in the AA LOAD subjects. These two variants are in tight LD with a D’ of 0.98, but R^2^ of 0.25 due to different allele frequencies. Logistic regression analysis conditioned on either variant revealed a stronger influence of p.L211P, suggesting that this variant is more likely to be functional than p.W191X. That said, p.W191X still has an estimated OR indicative of increased LOAD risk after conditioning on p.L211P, but was no longer significant.

We note that p.L211P is in tight LD with p.T96K, which was not genotyped in all cohorts because this locus is polyallelic (p.T96K/M/R). P.T96K resides within exon 2 where all previously reported *TREM2* disease risk variants are localized. Given these and the fact that p.T96K has a predicted damaging functional outcome; we recognize that the AD risk association observed by p.L211P may be driven by this variant. Collectively, our findings are consistent with a model where p.L211P, or p.T96K, is a stronger functional LOAD risk variant, with p.W191X also harboring a smaller but independent effect on LOAD risk. It will also be important to consider an alternative model, where p.L211P is in LD with several functional variants, including p.W191X and/or p.T96K, thus leading to enhanced risk estimates for p.L211P. In this alternative scenario, the other functional risk variants would be non-coding variants that would not be identified in our sequencing paradigm, rarer coding variants not tested in our study or a combination of both. Finally, it should be noted that p.W191X is in LD with the strongest *TREM2* region variant observed in a LOAD risk GWAS of AAs (rs7748513: OR ± SE, 1.16 ± 0.05, P = 0.001. LD between p.W191X and s7748513 estimated in the 274 Mayo Clinic samples that were included in the AA GWAS was D’ = 1, R^2^ = 0.06) [22]. Given that rs7748513 is deep within intron 2 of *TREM2*, the observed association in the AA GWAS is likely a consequence of tagging functional coding variants.

*TREM2* p.L211P resides within exon 4 of ENCODE transcript ID ENST00000373113 (RefSeq NM_018965), which encodes the full length TREM2 protein. Exon 4 is lacking from the shorter *TREM2* transcripts (ENST00000373122 which is a predicted protein and ENCODE ID ENST00000338469, a.k.a. RefSeq NM_001271821 or TREM-2V; See Figure 1). ENCODE ID ENST00000338469 encodes a shorter form of TREM2 (sTREM2) that includes the first 143 of the 156 amino acids within the predicted secreted extracellular domain, in addition to exon 5 that partially encodes the cytoplasmic domain and an additional 54 amino acid C-terminal domain not shared with the full length protein. *TREM2* p.W191X resides within this unique domain of TREM-2V and leads to a stop-gain mutation resulting in a predicted truncated TREM-2V. Theoretically, both of these coding changes could have consequences on the function of TREM2 via a number of mechanisms.

TREM2 is a type-I transmembrane protein, the extracellular domain of which was shown to undergo proteolytic processing via an ADAM or MMP protein family member [23] and in a more recent report, by ADAM10 [24], followed by intramembranous cleavage of its C-terminal fragment (CTF) by γ-secretase, which also cleaves amyloid precursor protein (APP) (reviewed [25]). Disruption of γ-secretase cleavage of TREM2 leads to accumulation of TREM2-CTFs, which in turn trap the TREM2 signalling adaptor protein DAP12, ultimately leading to reduced signaling [23]. Hence, a conformational change in TREM2, which alters cleavage by γ-secretase, may have downstream functional consequences. It will be important to test whether *TREM2* p.L211P which resides within the predicted cytoplasmic domain of the full length TREM2 alters its γ-secretase cleavage.

In a recent *in-vitro* study of TREM2 coding variants that reside within the extracellular domain, the FTD and FTD-like syndrome risk variants were shown to reduce maturation of TREM2, resulting in reduced cell surface expression, lower sTREM2 release extracellularly and lower phagocytic activity [24]. Thus, whether p.L211P or p.T96K may also alter TREM2 maturation with similar downstream consequences is another potential mechanism that requires evaluation. It is plausible that sTREM2 *per se* can have functions in signaling [24] or the immune system [26]. Therefore, the effects of p.L211P, p.T96K and p.W191X on sTREM2 levels merit investigation. Finally, p.W191X may reduce sTREM2 protein levels via nonsense-mediated mRNA decay. The missense mutations p.T96K and p.L211P could theoretically result in reduced protein levels via inefficient translation or degradation of full length TREM2, as previously demonstrated for missense mutations in other genes [27].

One caveat of this study is that since we sequenced a relatively small number of individuals and only screened for exon 2 in the Mayo control samples, it is possible that some potential functional variants (e.g. p.P3T, p.R5W, p.F170L, p.A192T, p.D216Y, and p.T223I based on NHLBI Exome Variant Server [EVS] data base) could have been missed in this study. However, since the aforementioned variants are extremely rare (range from 0.02% to 0.11% according to EVS; see Additional file 6: Table S13) in African Americans (AAs), our study lacks the statistical power to reach any meaningful conclusions for their association with AD risk. A larger-scale sequencing study of the *TREM2* region in AAs is needed to further determine the underlying variants driving the association with AD.

In our study of AA subjects we did not find a significant LOAD risk association with p.R47H, the most widely replicated *TREM2* LOAD risk variant in Caucasians, or with the additional risk variants p.R62H or p.D87N [6,7]. However, p.R47H and p.D87N are more frequent in AA cases compared to controls, as would be expected. The lack of association of these variants may be due to their very low MAF in AAs. All three of these variants are rarer in AA vs. EA subjects (EVS AA and EA MAFs are p.R47H: 0.02% vs. 0.26%, p.R62H 0.27% vs. 1%, and p.D87N 0% vs. 0.14%, respectively). Given this, our cohort was likely underpowered to detect any effects from these variants. Another possibility is that since these *TREM2* variants are risk factors rather than deterministic mutations, AA subjects may be lacking additional genetic and/or environmental factors, which exist in EAs and which may potentiate the effects of these variants on LOAD risk. Besides limited power, another weakness of our study is the lack of autopsy-confirmation, which is a universal problem with most LOAD case–control studies, but especially those conducted in non-Caucasian populations.

## Conclusions

In summary, our investigation of *TREM2* coding variants in AA subjects identified variants which confer LOAD risk, although the most strongly associated variants were not previously demonstrated as risk factors in Caucasian studies because they are much rarer in Caucasian samples. Our study has several strengths including variant discovery through deep-sequencing of two independent cohorts via different approaches, validation by genotyping of a relatively large combined LOAD case–control series from five centers, case–control association analyses and a careful analytic paradigm to discern independent effects of the top two variants. Our findings highlight the importance of investigating different ethnic populations, which may lead to discoveries of novel risk variants due to allelic heterogeneity. The strongest variants identified in our study appear to implicate different regions (cytoplasmic domain) and transcripts (TREM-2V) of TREM2 in LOAD risk than prior reports (exon 2/extracellular domain) in Caucasians. Although the functional consequences of these variants remain to be established, such findings pave the way to inquire alternative disease risk mechanisms and may therefore further our understanding of the role of TREM2 in LOAD and other neurodegenerative diseases.

## Methods

**Figure 2:**
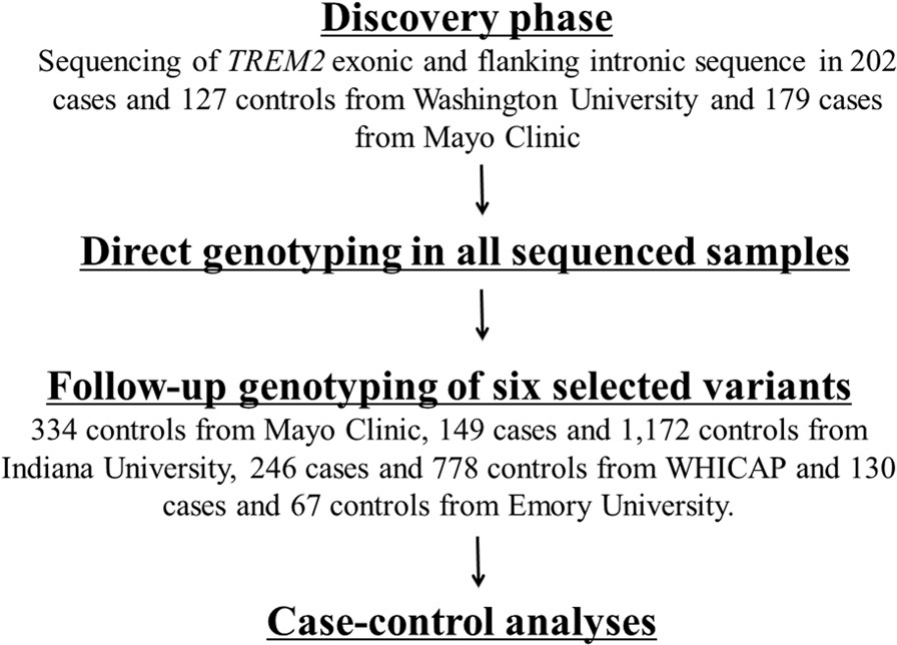
**Study design for assessing** ***TREM2*** **association with AD risk in African Americans.**

In order to maximize the statistical power in detecting significant rare-variant association, we analyzed DNA samples from AA individuals evaluated at Washington University School of Medicine in St. Louis (WUSM; includes samples from Knight-Alzheimer’s Disease Research Center [Knight-ADRC] and NIA-LOAD), Mayo Clinic Jacksonville in Florida (Mayo), Indiana University School of Medicine (Indiana), Washington Heights-Inwood Community Aging Project (WHICAP), and the Emory University (Emory). All of these individuals were self-reported AAs. Characteristics of the individual cohorts are listed in Table 2. The overall study design is depicted in Figure 2. Further details are provided in the Additional file 7: Supplementary methods and Additional file 8: Tables S1-S6.

**Table 2:** **Demographic information of studied African Americans**

**Cohort**	**No. of participants**	**Type**	**Sequencing/Genotyping strategy**	**Cases**		**Controls**	
				**N**	**Range of age at onset/diagnosis**	**N**	**Range of age at last assessment**
Knight-ADRC + NIA-LOAD	338	Clinical	Pooled sequencing/Sequenom + KASPar	202	60-94	136	60-97
Mayo Clinic	513	Clinical	Sanger sequencing (exons 1–5 in AD cases and exon 2 in controls)/Taqman + KASPar	179	61-99	334	60-98
Indiana University	1,321	Clinical	Sequenom + KASPar	149	72-102	1,172	70-102
WHICAP	1,024	Clinical	KASPar	246	68-101	778	66-99
Emory University	197	Clinical	Taqman + KASPar	130	61-98	67	60-94*

Type: Type of Alzheimer’s disease patients. *Range of age at draw.

## Additional files

Additional file 1: Tables S7-S8.
*TREM2* variants discovered and validated in African Americans. Tables show the variants discovered and validated in the **(Table S7).** Washington University in St. Louis and **(Table S8).** Mayo Clinic Florida cohorts. NA represents not applicable. The Fisher’s exact test was used to calculate p-values and odds-ratio (OR) for each variant using the default commands in PLINK. Multivariate logistic regression was performed to evaluate the association of p.T96K, p.W191X and p.L211P with AD risk adjusting for age, gender, and *APOE* genotype. Additional file 2: Tables S9-S11.
*TREM2* variants genotyped in African Americans. Tables show the variants genotyped at **(Table S9).** Indiana University **(Table S10).** WHICAP, and **(Table S11).** Emory University. NA represents not applicable. The Fisher’s exact test was used to calculate p-values and odds-ratio (OR) for each variant using the default commands in PLINK. Multivariate logistic regression was performed to evaluate the association of p.W191X and p.L211P with AD risk adjusting for age, gender, and *APOE* genotype. Additional file 3: Figure S1. Forest plot for p.W191X odds ratios across cohorts. Forest plot of multivariate logistic regression results generated using the R package ‘rmeta’. Additional file 4: Figure S2. Forest plot for p.L211P odds ratios across cohorts. Forest plot of multivariate logistic regression results generated using the R package ‘rmeta’. Additional file 5: Table S12. Conditional analyses for p.W191X and p.L211P. Conditional association testing was performed to evaluate the association of p.W191X and p.L211P with AD risk adjusting for age, gender, *APOE* genotype, cohort, and the conditioning SNP. Additional file 6: Table S13.
*TREM2* variants that are present in the African Americans according to NHLBI EVS. The amino acid change, exon location, dbSNP reference number, GVS function category, minor allele frequency (MAF) in 2,203 African Americans, and PolyPhen2 functional prediction of TREM2 variants are listed in each row. The amino acid position is annotated based on the longest protein isoform. &Rs2234258 (p.W191X) is only present in the coding region of the shortest isoform of TREM2 (ENST00000338469). Variants observed in the in 202 AD cases and 136 controls sequenced at Washington University, or in the 179 AD cases sequenced at Mayo Clinic, are shown in bold font. EVS does not list 5 of the missense variants (p.T66M, p.D87N, p.T96M, p.A105V, and p.E202D) that were observed in our study sample. Additional file 7:
**Supplementary Methods.** Extended methods detailing the design, cohorts, and statistical methods applied. Additional file 8: Tables S1-S6. Demographic characteristics of African Americans. Tables detail the demographic characteristics of the various cohorts. Sample size (N), mean, standard deviation and range for age in years, percentage of female subjects and percentage of subjects that carry at least one *APOE*-ε4 allele for the AD cases and cognitively normal elderly controls from **(Table S1).** Knight-ADRC, **(Table S2).** NIA-LOAD, **(Table S3).** Mayo Clinic, **(Table S4).** Indiana University, **(Table S5).** WHICAP, **(Table S6).** Emory University. Ages are age-at-onset for ADs and age of last evaluation for controls.

## Abbreviations

TREM2: Triggering receptor expressed on myeloid cells 2
PLOSL: Polycystic lipomembranous osteodysplasia with sclerosing leukoencephalopathy
AD: Alzheimer’s disease
FTD: Frontotemporal dementia
AA: African-Americans
LOAD: Late-onset Alzheimer’s disease
SNP: Single nucleotide polymorphism
WUSM: Washington University School of Medicine in St. Louis
WHICAP: Washington Heights-Inwood Community Aging Project
OR: Odds ratio
LD: Linkage disequilibrium
EA: European American
MAF: Minor allele frequency
ESP: Exome Sequencing Project
EVS: Exome Variant Server
GWAS: Genome-wide association study
ADAM: A disintegrin and metalloproteinase
MMP: Matrix metalloproteinase
CTF: C-terminal fragment
APP: Amyloic precursor protein
