# Supplementary material for: TREM2 is associated with increased risk for Alzheimer’s disease in African Americans
Source: Mol Neurodegener. 2015 Apr 10;10:19. doi: 10.1186/s13024-015-0016-9 (PMC4426167; doi:10.1186/s13024-015-0016-9)
Supplement: Additional file 3: Figure S1. — Forest plot for p.W191X odds ratios across cohorts. Forest plot of multivariate logistic regression results generated using the R package ‘rmeta’. [file 13024_2015_16_MOESM3_ESM.docx]

**
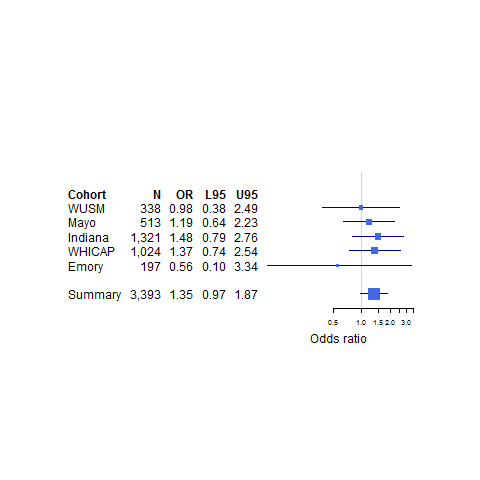
**

**Figure S1. Forest plot for p.W191X odds ratios across cohorts.**

Forest plot of multivariate logistic regression results generated using

the R package 'rmeta'.
