# Supplementary material for: TREM2 is associated with increased risk for Alzheimer’s disease in African Americans
Source: Mol Neurodegener. 2015 Apr 10;10:19. doi: 10.1186/s13024-015-0016-9 (PMC4426167; doi:10.1186/s13024-015-0016-9)
Supplement: Additional file 4: Figure S2. — Forest plot for p.L211P odds ratios across cohorts. Forest plot of multivariate logistic regression results generated using the R package ‘rmeta’. [file 13024_2015_16_MOESM4_ESM.docx]

**
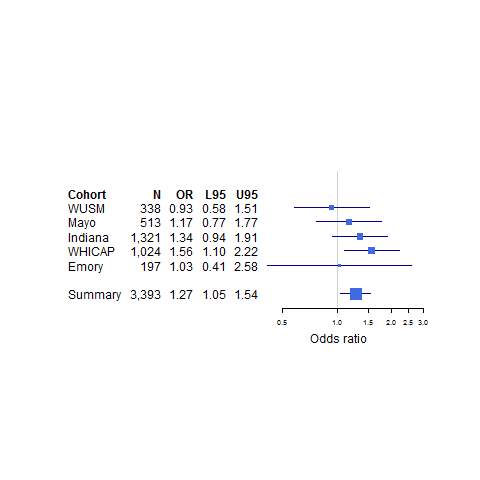
**

**Figure S2. Forest plot for p.L211P odds ratios across cohorts.**

Forest plot of multivariate logistic regression results generated using

the R package 'rmeta'.
