# Supplementary material for: TREM2 is associated with increased risk for Alzheimer’s disease in African Americans
Source: Mol Neurodegener. 2015 Apr 10;10:19. doi: 10.1186/s13024-015-0016-9 (PMC4426167; doi:10.1186/s13024-015-0016-9)
Supplement: Additional file 7: — Supplementary Methods. Extended methods detailing the design, cohorts, and statistical methods applied. [file 13024_2015_16_MOESM7_ESM.docx]

**Supplementary Methods**

**Sample collection, study design, and data quality control**

In our discovery phase, deep re-sequencing was performed for the exonic and flanking intronic sequence of *TREM2* in 329 AA subjects from WUSM (202 LOAD and 136 controls) and 179 LOAD cases from Mayo. Additionally, 334 controls from Mayo underwent Sanger sequencing for *TREM2* exon 2 region. To validate called variants, we then performed direct genotyping in all sequenced individuals totaling 338 subjects from WUSM and 513 from Mayo. We selected six potentially functional *TREM2* variants (p.R47H, p.R62H, p.D87N, p.E151K, p.W191X, and p.L211P) for follow-up genotyping in samples from Indiana, WHICAP, and Emory. These six variants were all non-synonymous changes that were observed at least once in one of the sequenced cohorts from WUSM and Mayo. Detailed rationale for their selection is provided in the Results.

Stringent quality control (QC) filters were adopted to exclude individuals from final analyses. An individual was excluded if he/she met either of the following criteria: (1) age at onset or age at last assessment is below 60 years, (2) more than one missing genotype out of the six selected variants. After QC, a total of 3 393 individuals (338 from WUSM, 513 from Mayo, 1,321 from Indiana, 1,024 from WHICAP, and 197 from Emory) were included in the final analyses (**Table 1**).

**Washington University Knight-ADRC Cohort:**

These samples were collected at the Knight-ADRC at Washington University and were evaluated by the Clinical Core of the Knight-ADRC. LOAD cases received a clinical diagnosis of AD dementia in accordance with National Institute of Neurological and Communication Disorders and Stroke-Alzheimer's Disease and Related Disorders Association (NINCDS-ADRDA) criteria [[1](#_ENREF_1)], and dementia severity was determined with the Clinical Dementia Rating (CDR) [[2](#_ENREF_2)], with higher scores being associated with more severe cognitive decline. Controls underwent the same assessment and were cognitively normal. The Knight-ADRC samples were recruited without enrichment based on family history.

The Institutional Review Board (IRB) at the Washington University School of Medicine in Saint Louis approved the study. A written informed consent was obtained from participants and their family members by the Clinical Core of the Charles F. and Joanne Knight Alzheimer’s Disease Research Center (Knight-ADRC). The approval number for the Knight-ADRC Genetics Core family studies is 93-0006.

Targeted next-generation sequencing of pooled-DNA was performed for each exonic and flanking sequence in *TREM2* in 150 LOAD cases and 93 cognitively normal controls of AA descent (**Table S1**) as described previously [[3-6](#_ENREF_3)]. Direct genotyping was performed in all sequenced individuals using either Sequenom iPLEX or KASPar genotyping systems.

**Washington University NIA-LOAD Cohort:**

All AD cases were diagnosed with dementia of the Alzheimer's type (DAT) using criteria equivalent to the NINCDS-ADRDA for probable AD. All NIA-LOAD AD cases have a family history of AD. Probands were required to have a diagnosis of definite or probable AD and a sibling with definite, probable or possible AD with a similar age at onset. A third biologically-related family member (first, second or third degree) was also recruited, regardless of cognitive status. For this study we used one affected individual per family, selecting the youngest affected family member with the most definitive diagnosis (i.e. individuals with autopsy confirmation were chosen over those with clinical diagnosis only). Unrelated controls were cognitively normal and had no family history of dementia.

Written informed consent was obtained from all participants, and the study was approved by local IRB committees.

We performed pooled sequencing in this NIA-LOAD case-control series of AA descent, which included 52 AD cases and 43 healthy unrelated controls (**Table S2**). We used the Sequenom iPLEX or KASPar genotyping systems to confirm called variants.

**Mayo Clinic Cohort**

Subjects included in this cohort (179 LOADs vs. 334 controls) were self-described AAs, recruited at Mayo Clinic Jacksonville in Florida. All subjects were evaluated by a neurologist. Controls were cognitively normal at last visit with Clinical Dementia Rating scale (CDR) = 0, while cases had a diagnosis of possible or probable AD made according to NINCDS-ADRDA criteria [[1](#_ENREF_1)]. Summary demographics for the Mayo Clinic African American LOAD case-control series are shown on **Table S3.**

Informed consent was obtained from all study participants and this study was approved by the Mayo Clinic IRB.

DNA extracted from blood of Mayo AA LOAD patients was used to identify *TREM2* coding variants in exons 1-5, and in controls in exon 2 only, via Sanger sequencing on an ABI 3730 automated sequencer (Applied Biosystems, California, USA). Primer sequences are available upon request. The sequence data was analyzed with Sequencher® 4.8 (Gene Codes Corporation, Michigan, USA) and compared to the published reference sequence for *TREM2* transcripts (Refseq NM_018965 and NM_00127182, human genome assembly GRCh37). Genotyping was done using TaqMan® SNP Genotyping Assays in an ABI PRISM® 7900HT Sequence Detection System with 384-Well Block Module from Applied Biosystems, California, USA. The genotype data was analyzed using the SDS software version 2.2.3 (Applied Biosystems, California, USA). Due to the presence of another variant within the Taqman assay design, the p.R62H SNP was genotyped using a KASPar assay (LCG Genomics, Beverly, Massachusetts, USA); and the genotype data was also analyzed using the SDS software version 2.2.3 (Applied Biosystems, California, USA). Genotype assay information is available upon request.

**Indiana University Cohort**

Subjects included as part of the Indiana cohort were diagnosed according to NINCDS-ADRDA criteria and have been previously described [[7](#_ENREF_7)]. See **Table S4** for the demographic information.

Informed consent was obtained from all study participants and the study was approved by the Indiana University IRB.

Direct-genotyping of six variants in 149 AD cases and 1,172 controls of AA descent from Indiana University School of Medicine was performed using Sequenom iPLEX or KASPar genotyping system.

**WHICAP Cohort**

The WHICAP participants were part of a longitudinal cohort study enrolled by a random sampling of Medicare recipients 65 years or older residing in northern Manhattan, New York [[8](#_ENREF_8)]. Each participant underwent an interview of general health and function, medical history, a neurological examination, and a neuropsychological battery. Baseline data were collected from 1999 through 2001. Follow-up data were collected at sequential intervals of 18 months. Diagnosis of dementia etiology was made based on NINCDS-ADRDA criteria and severity of dementia was assessed using the Clinical Dementia Rating scale. See **Table S5** for detailed demographic information. Written informed consent was obtained from all participants, and the study was approved by local IRB committees.

Six variants were genotyped in 246 AD cases and 778 controls of AA descent from WHICAP using the KASPar genotyping system.

**Emory University Cohort**

At Emory University, six variants were genotyped using Taqman SNP Genotyping Assays, or the KASPar genotyping system, in 130 AD cases and 67 controls that self-identified as AA. See **Table S6** for demographic information. Informed consent was obtained from all study participants and the study was approved by the Emory University IRB.

**Genotyping quality control**

*TREM2* missense variants found via sequencing in the Mayo samples were genotyped with either Taqman or KASPar assays in both the Mayo AD cases and control samples, achieving greater than 99% concordance rate between the sequencing and Taqman or KASPar genotypes. All other genotypes were generated using a single genotyping technique.

**Statistical and bioinformatics analyses:**

Association of genetic variants with AD risk was assessed using the Fisher’s exact test implemented in PLINK [[9](#_ENREF_9)] ( <http://pngu.mgh.harvard.edu/purcell/plink/>). Multivariate logistic regression analysis of the two common variants (p.W191X and p.L211P) were also performed in PLINK adjusting for age, gender, *APOE* ε4 dosage and cohort. Age is defined as age at onset (WUSM and WHICAP) or diagnosis (Mayo and Indiana) for AD patients, and age at last clinical evaluation for control subjects, except for the Emory cohort where age at blood draw was used for both. SIFT and Polyphen-2 functional predictions were obtained from the respective websites (<http://sift.jcvi.org/> and <http://genetics.bwh.harvard.edu/pph2/bgi.shtml>, respectively). The Uniprot database (<http://www.uniprot.org/>) was used to extract and perform alignment of the protein sequences across different species.

**References**

1. McKhann G, Drachman D, Folstein M, Katzman R, Price D, Stadlan EM. Clinical diagnosis of Alzheimer's disease: report of the NINCDS-ADRDA Work Group under the auspices of Department of Health and Human Services Task Force on Alzheimer's Disease. Neurology. 1984;34(7):939-44.

2. Morris JC. The Clinical Dementia Rating (CDR): current version and scoring rules. Neurology. 1993;43(11):2412-4.

3. Jin SC, Benitez BA, Karch CM, Cooper B, Skorupa T, Carrell D et al. Coding variants in TREM2 increase risk for Alzheimer's disease. Human molecular genetics. 2014;23:5838-46. doi:10.1093/hmg/ddu277.

4. Cruchaga C, Chakraverty S, Mayo K, Vallania FL, Mitra RD, Faber K et al. Rare variants in APP, PSEN1 and PSEN2 increase risk for AD in late-onset Alzheimer's disease families. PLoS One. 2012;7(2):e31039. doi:10.1371/journal.pone.0031039.

5. Jin SC, Pastor P, Cooper B, Cervantes S, Benitez BA, Razquin C et al. Pooled-DNA sequencing identifies novel causative variants in PSEN1, GRN and MAPT in a clinical early-onset and familial Alzheimer's disease Ibero-American cohort. Alzheimers Res Ther. 2012;4(4):34. doi:10.1186/alzrt137.

6. Benitez BA, Karch CM, Cai Y, Jin SC, Cooper B, Carrell D et al. The PSEN1, p.E318G variant increases the risk of Alzheimer's disease in APOE-epsilon4 carriers. PLoS genetics. 2013;9(8):e1003685. doi:10.1371/journal.pgen.1003685.

7. Hendrie HC, Baiyewu O, Lane KA, Purnell C, Gao S, Hake A et al. Homocysteine levels and dementia risk in Yoruba and African Americans. Int Psychogeriatr. 2013;25(11):1859-66. doi:10.1017/S1041610213001294.

8. Tang MX, Stern Y, Marder K, Bell K, Gurland B, Lantigua R et al. The APOE-epsilon4 allele and the risk of Alzheimer disease among African Americans, whites, and Hispanics. Jama. 1998;279(10):751-5.

9. Purcell S, Neale B, Todd-Brown K, Thomas L, Ferreira MA, Bender D et al. PLINK: a tool set for whole-genome association and population-based linkage analyses. Am J Hum Genet. 2007;81(3):559-75. doi:10.1086/519795.
